# A nationwide exploratory survey assessing perception, practice, and barriers toward pharmaceutical care provision among hospital pharmacists in Nepal

**DOI:** 10.1038/s41598-022-16653-x

**Published:** 2022-10-05

**Authors:** Rajeev Shrestha, Subish Palaian, Binaya Sapkota, Sunil Shrestha, Asmita Priyadarshini Khatiwada, Pathiyil Ravi Shankar

**Affiliations:** 1 Department of Pharmacy, District Hospital Lamjung, Lamjung, Gandaki Nepal; 2 Present Address: Department of Clinical Sciences, College of Pharmacy and Health Sciences, Ajman University, Ajman, United Arab Emirates; 3 Department of Pharmaceutical Sciences, Nobel College, Affiliated to Pokhara University, Kathmandu, Bagmati Nepal; 4 School of Pharmacy, Monash University Malaysia, Jalan Lagoon Selatan, 47500 Bandar Sunway, Selangor Malaysia; 5 Department of Pharmaceutical and Health Service Research, Nepal Health Research and Innovation Foundation, Lalitpur, Bagmati Nepal; 6 IMU Centre for Education, International Medical University, Kuala Lumpur, Malaysia

**Keywords:** Health care, Health services, Public health

## Abstract

Pharmaceutical care (PC) services reduce medication errors, improve the use of medicines, and optimize the cost of treatment. It can detect medication-related problems and improve patient medication adherence. However, PC services are not commonly provided in hospital pharmacies in Nepal. Therefore, the present study was done to determine the situation of PC in hospital pharmacies and explore the perception, practice, and barriers (and their determinants) encountered by hospital pharmacists while providing PC. A descriptive online cross-sectional study was conducted from 25th March to 25th October 2021 among pharmacists with a bachelor’s degree and above working in hospital pharmacies using non-probability quota sampling. The questionnaire in English addressed perception and practice regarding PC, and barriers encountered and were validated by experts and pre-tested among 23 pharmacists. Descriptive statistics were used to describe the data. Kendall’s correlation was used to explore the correlations among various perception and practice constructs. The scores were also compared among subgroups of respondents using the Mann–Whitney test for subgroups with two categories and Kruskal–Wallis test for greater than two categories. A total of 144 pharmacists participated in the study. Majority of the participants were male, between 22 and 31 years of age, and had work experience between 10 and 20 years. Over 50% had received no training in PC. The perception scores were higher among those with more work experience and the practice scores among those who had received PC training. Participants agreed that there were significant barriers to providing PC, including lack of support from other professionals, lack of demand from patients, absence of guidelines, inadequate training, lack of skills in communication, lack of compensation, problems with access to the patient medical record, lack of remuneration, and problems with accessing objective medicine information sources. A correlation was noted between certain perceptions and practice-related constructs. Hospital pharmacists who participated had a positive perception and practice providing PC. However, PC was not commonly practised in hospital pharmacies. Significant barriers were identified in providing PC. Further studies, especially in the eastern and western provinces, are required. Similar studies may be considered in community pharmacies.

## Introduction

Medicines are an important part of healthcare services provided to the users by a team of healthcare professionals, including doctors, pharmacists, nurses, and others. Medicine-related errors are common in Nepal while providing these services^1–3^. Medicine-related errors eventually affect the quality of medical care and worsen the health-related quality of life (HRQoL) (increased morbidity and mortality rate), economy, and life expectancy^4–6^. Therefore, rational use of medicines and medication error prevention is a pressing need to reduce the health-related financial burden and preserve and promote HRQoL. This can be achieved to a larger extent by providing pharmaceutical care. Pharmaceutical Care (PC), as defined by Hepler and Strand (1990), “*is the responsible provision of drug therapy for the purpose of achieving definite outcomes that improve a patient’s quality of life that involves the process through which a pharmacist co-operates with a patient and other professionals in designing, implementing and monitoring a therapeutic plan that will produce specific therapeutic outcomes for the patient*”^7^. Pharmaceutical Care Network Europe (PCNE, 2013) defines PC as “*the pharmacist’s contribution to care of individuals in order to optimize medicines use and improve health outcomes*”^8^.

PC helps prevent disease complications through early identification, detection, and prevention of medicine-related problems, improves patient medication adherence, achieves therapeutic objectives, and makes the public aware of healthy lifestyle choices^9^. Major objectives of PC include identifying and mitigating pharmacotherapy-related problems by pharmacists in collaboration with other healthcare providers (e.g., clinicians, nurses)^10^. Many studies globally have reported improved health outcomes, reduced economic burden, and rational medication use through the provision of PC in patients with various disease conditions such as diabetes^11^, cardiovascular diseases^12^, and chronic respiratory diseases^13^. PC provision is important to mitigate the COVID-19 pandemic as well^14,15^. Pharmacists have played a pivotal role in providing medicine-related services and PC in many countries^15–18^.

The pharmacy profession is still not well developed in Nepal, and pharmacists working in hospital settings are mainly engaged in dispensing, counselling about dispensed medicines, procurement, and managing pharmaceuticals and surgical items^19^. Although the clinical role of pharmacists is emerging in Nepal^19,20^, PC services are being provided only in very few hospitals where patients may be satisfied with the services^21,22^. Pharmacists provide clinical services, including drug information services, pharmacovigilance services, medication counselling, and patient education within the health facilities^19,22–25^.

Since many pharmacies in Nepal are still run by unqualified persons^26,27^, the status of PC in actual practice is questionable. To enhance the quality and standard of overall pharmaceutical service by pharmacists, the Nepal Pharmacy Council (NPC) developed the National Good Pharmacy guideline draft in 2005. In addition, the Department of Drug Administration (DDA), Nepal, developed a ‘Hospital Pharmacy Service Directive’ in 2015. Nevertheless, the implementation of these guidelines is lacking. The pharmacy undergraduate students of Nepal are deemed to understand the concept of PC and have a positive attitude towards its practice. However, various challenges include insufficient training and education on PC, constraints in obtaining patients’ clinical files with usual manual documentation practice, lack of drug information resources in pharmacies, and space problems in pharmacies located within the premises of private and government hospitals^28^.

Moreover, there is paucity of studies reporting perception, practice, and barriers regarding PC provision from the pharmacists’ point of view. So, the current study aimed to determine the situation of PC, and explore the perception, practice, and barriers (and their determinants) encountered by hospital pharmacists while providing PC in Nepal. This study would also provide a background for the concerned regulatory bodies to devise policies and arrangements to improve the PC services in Nepal.

## Method

### Study design and study period

A descriptive cross-sectional questionnaire-based survey was conducted from 25th March to 25th October 2021 in Nepal.

### Study setting

The study was carried out among pharmacists working in hospital pharmacies in all seven provinces of Nepal, a lower middle-income country (LMIC) in South Asia. Pharmacists included in the study were those with a Bachelor’s degree in pharmacy (BPharm) or above. According to the pharmaceutical country profile, 2017, there are 0.8 registered pharmacists per 10,000 population in Nepal^29^.

### Sample size

The sample size was calculated as 207 using the online survey size calculator considering a 95% confidence level and 5% confidence interval^30^. As the total population of pharmacists working in hospital pharmacies in Nepal was unknown, the target population was estimated using the International Pharmaceutical Federation (FIP) data of pharmacists working in hospital pharmacies in LMICs and NPC data on total registered pharmacists in Nepal. According to FIP and NPC, 9.3% of pharmacy practitioners work in hospital pharmacy settings in LMICs^30^, and 4829 pharmacists were registered in Nepal on 25th March 2020^31^. These together provide a tentative population (449) of hospital pharmacists for sample size calculation. Adding 10% non-response, a total sample size of 228 (207 + 21) was calculated.

### Sampling procedure

Non-probability quota sampling method was used, and the estimated sample was divided among all seven provinces based on the distribution of healthcare facilities to obtain the nationwide proportional representation^32^. Pharmacists working in hospital pharmacies were conveniently selected based on professional networks and interest in participating. Pharmacists with a bachelor’s degree in pharmacy or above and registered with the NPC were included. However, assistant pharmacists, pharmacy students, medical representatives, and industrial pharmacists were excluded. The estimated sample size is presented in Table 1.

**Table 1:** Distribution of pharmacists among the seven provinces.

Provinces of Nepal	Total no of health facilities		Division of sample proportionately
	Number	Proportion (%)	
Province 1*	952	13.7	32
Madhesh	991	14.3	32
Bagmati	2320	33.5	75
Lumbini	736	10.6	25
Gandaki	915	13.2	30
Karnali	464	6.7	16
Sudurpashchim	556	8.0	18
Total	6934	100	228

*No name has been given to province 1 yet, as of 23rd April, 2022.

### Ethical approval and informed consent

The ethical approval was obtained from the Ethical Review Board of Nepal Health Research Council (NHRC), Kathmandu, Nepal, on 16th May 2021 (Ref Number: 3136). Participants were informed about the study, and written informed consent was also obtained before collecting the responses. All methods applied were performed in accordance with the relevant guidelines and regulations.

## Data collection tools

### Development of the structured questionnaire

The questionnaire was designed as a self-administered tool. The draft was initially prepared after an extensive literature review^28,33–48^ and a thorough discussion among the co-authors. The authors who created the initial draft were hospital pharmacists, clinical pharmacists, and academicians in pharmacy. The tool was developed in English.

The data collection tool consisted of four parts: patient’s demographic and work-related information, perception-related questions, practice-related questions, and barrier-related questions. Initially, the socio-demographic and work-related section, perception-related section, practice-related section, and barrier-related section consisted of 11, 9, 11 and 27 questions, respectively. There were higher number of barrier-related questions since the researchers were interested in a more in-depth assessment of barriers. In addition, the researchers assumed that there were more barriers to PC in Nepal, which has prevented establishing and providing the same efficiently in hospital pharmacies.

### Data collection

A web-based online approach was used to collect responses from the pharmacists at various hospital pharmacies. The online survey link for data collection was shared through pharmacy professional associations, email, online pharmacy networking portals/groups on social media, and WhatsApp groups. The first page of the survey contained the objective, nature, and benefit of the study which is followed by agree/disagree option at the end of the first page. Those who consented and agreed to participate in the study navigated to the questionnaire page on clicking the agree button.

### Content validity

A panel of four experts was selected for the face and content validation of the research instrument. The panel consisted of university professors, lecturers and PhD scholars residing both in Nepal and abroad. They reviewed the questionnaire and provided their insights on the understanding of items and completeness of the questionnaire for measuring each theme and suggestions for revision. As per the experts’ suggestion, the arrangement, language, terminology, and question structure were revised, such as ‘lack of motivation’ was changed into ‘pharmacist lack motivation’, ‘Keeping patient’s clinical and medical information record’ into ‘Documenting patient’s clinical and medication information record’.

### Face validity

The face validity of the questionnaire was studied among 23 pharmacists (10% of the total estimated sample) working in the province Bagmati. Participants were asked to complete the questionnaire and comment on its ease of understanding, readability, clarity, and suitability. The comments and suggestions received from the participants were discussed among the authors, and the tool was finalized. The data from these respondents were not included in the final analysis. After validation and reliability testing, patient’s demography and work-related questions, perception-related questions, practice-related questions, and barrier-related sections consisted of 14, 6, 11 and 26 questions, respectively (see S1 File).

### Reliability analysis

Cronbach’s alpha value was calculated. It was found to be 0.429, 0.832 and 0.872 for perception-related questionnaires, practice-related questionnaires, and barrier-related questionnaires, respectively. As the alpha value for the perception-related questionnaire was low, three questions were removed following discussion among co-authors and only six were retained, giving an alpha value of 0.602.

### Data analysis

Data from the predesigned form was entered in MS Excel and then analyzed with IBM statistical package for social sciences (SPSS) v 26.0 (SPSS Inc., Chicago, IL, USA) by applying descriptive statistics (i.e., mean, SD, frequency, and percentage) and inferential statistics (Kendall’s correlation) to explore the correlations among various perception and practice constructs of pharmaceutical care with qualifications, experience, site of work, working hours of the pharmacists, age of the respondents and number of daily prescriptions handled (i.e., daily workload). Values of Kendall’s tau were interpreted as less than ± 0.25 (very weak), ± 0.25 to ± 0.34 (weak), ± 0.35 to ± 0.39 (moderate), and ± 0.40 or larger (strong relationship). In addition, perception and practice scores were compared among subgroups using the Mann–Whitney U-test for two categories and the Kruskal–Wallis test for more than two categories. A p value < 0.05 was considered statistically significant at a 95% confidence interval.

## Results

### Demographic characteristics of study participants

A total of 144 pharmacists responded. Maximum were male pharmacists (90, 62.5%), aged 22–31 years (119, 82.6%), with Bachelor’s in pharmacy (BPharm) degree (109, 75.7%), work experience of 10.1–20.0 years (133, 92.4%), and working at a private hospital pharmacy (65, 45.1%). More participants (143, 99.3%) worked on average for more than 35 h per week. Of the respondents, 76 (52.8%) participants had not received training in providing pharmaceutical care (Table 2).

**Table 2:** Demographic characteristics of study participants (n: 144).

Study variables	Frequency (%)
**Age (in years) (mean ± SD: 28.18 ± 4.29)**	
≤ 21	1 (0.7)
22–31	119 (82.6)
32–41	23 (16)
52+	1 (0.7)
**Gender**	
Male	90 (62.5)
Female	54 (37.5)
**Qualification**	
MPharm	20 (13.9)
PharmD	15 (10.4)
BPharm	109 (75.7)
**Work experiences (in years) (Mean ± SD: 4.11 ± 3.97)**	
≤ 0	2 (1.4)
0.1–10.0	133 (92.4)
10.1–20.0	8 (5.6)
30.1+	1 (0.7)
**Training in pharmaceutical care**	
Not received	76 (52.8)
Received	68 (47.2)
**Current site of work**	
Government hospital pharmacy	58 (40.3)
Private hospital pharmacy	65 (45.1)
Community or NGO hospital pharmacy	21 (14.6)
**Working hours per week (mean ± SD: 46.72 ± 7.50)**	
≤ 34	1 (0.7)
35+	143 (99.3)
**Daily number of prescriptions handled (mean ± SD: 80.78 ± 82.64)**	
≤ 10	1 (0.7)
11–110	119 (82.6)
111–210	18 (12.5)
211–310	3 (2.1)
411+	3 (2.1)
**Provinces of Nepal**	
Province 1*	5 (3.5)
Madhesh	5 (3.5)
Bagmati	75 (52.08)
Lumbini	21 (14.6)
Gandaki	23 (16.0)
Karnali	5 (3.5)
Sudurpashchim	10 (6.9)

*Unnamed as of 5thdJune, 2022.

Unfortunately, we could not attain the estimated sample size from six provinces except Province Bagmati. This was because of various reasons, such as inability to physically reach every hospital amid the COVID-19 pandemic, a comparatively lower proportion of graduate pharmacists in hospital pharmacies outside the capital city of Nepal, which is located in the province Bagmati, and the unwillingness of pharmacists to enrol in the study. The detail of the location of the pharmacists who participated is given in the supplementary file (see S2 File).

### Pharmacy-related characteristics

Majority of the hospital pharmacies (101, 70.1%) operated more than 21 h daily, providing service to both outpatients and inpatients (125, 86.8%). An equal number of hospitals (41, 28.5%) had bed sizes of < 50 beds and ≥ 500 beds, and 95 (66%) pharmacies had 2–5 pharmacists in each shift (Table 3).

**Table 3:** Pharmacy-related characteristics.

Study variables	Frequency (%)
**Daily working hours of pharmacy (mean ± SD: 19.84 ± 6.58)**	
≤ 4	1 (0.7)
5–12	37 (25.7)
13–20	5 (3.5)
21+	101 (70.1)
**Services provision of pharmacy**	
Outpatients only	15 (10.4)
Both outpatients and inpatients	125 (86.8)
Inpatients only	4 (2.8)
**Bed size of the associated hospital (mean ± SD: 264.22 ± 261.49)**	
< 50 beds	41 (28.5)
50–99 beds	5 (3.5)
100–199 beds	30 (20.8)
200–299 beds	13 (9)
300–399 beds	11 (7.6)
400–499 beds	3 (2.1)
≥ 500 beds	41 (28.5)
**Number of pharmacists during each shift (mean ± SD: 3.22 ± 2.29)**	
≤ 1	31 (21.5)
2–5	95 (66)
6–9	13 (9)
10–13	4 (2.8)
14+	1 (0.7)

### Pharmacists’ perception regarding providing pharmaceutical care

Of the respondents, 86 (59.7%) strongly agreed that patient’s medications should be reviewed to prevent medicine-related error, and 92 (63.9%) strongly agreed that pharmaceutical care improves patient’s treatment or health outcomes (Table 4).

**Table 4:** Constructs related to perception regarding providing pharmaceutical care (n = 144).

Constructs	Frequency (%)					Median (IQR) scores
	Strongly disagree	Disagree	Not sure	Agree	Strongly agree	
1. Patient’s medications should be reviewed to prevent medicine-related errors and promote appropriate use of medications	4 (2.8)	2 (1.4)	–	52 (36.1)	86 (59.7)	5 (4–5)
2. All patients receiving medicines require pharmaceutical care services	5 (3.5)	7 (4.9)	10 (6.9)	67 (46.5)	55 (38.2)	4 (4–5)
3. Pharmaceutical care can improve patient’s treatment or health outcome	2 (1.4)	1 (0.7)	–	49 (34)	92 (63.9)	5 (4–5)
4. Pharmacists are professionally skilled health personnel in providing pharmaceutical care	11 (7.6)	3 (2.1)	2 (1.4)	49 (34)	79 (54.9)	5 (4–5)
5. Pharmacists are responsible for identification, prevention and resolution of medicine-related problems	3 (2.1)	1 (0.7)	1 (0.7)	61 (42.4)	78 (54.2)	5 (4–5)
6. Continuing pharmacy education is NOT essential to equip pharmacists to provide pharmaceutical care*	61 (42.4)	48 (33.3)	12 (8.3)	17 (11.8)	6 (4.2)	4 (4–5)

*The statement is negative and hence reversed while scoring.

### Current practice of pharmaceutical care

Maximum pharmacists (90, 62.5%) counselled patients to prevent potential drug therapy-related problems, but maximum number of pharmacist (12, 8.3%) confessed that they never does monitoring of adverse effects of medicine. Only 34 pharmacist (23.6%) reported of monitoring adverse effects of medicine all the time (Table 5).

**Table 5:** Constructs related to the current practice of pharmaceutical care (n = 144).

Constructs	Frequency (%)					Median (IQR) scores
	Never	Rare	Sometimes	Usually	All the time	
1. Enquiring about and reviewing patient’s medical and medicine records to decide if any intervention or recommendation must be made	2 (1.4)	16 (11.1)	42 (29.2)	44 (30.6)	40 (27.8)	4 (3–5)
2. Documenting patient’s clinical and medication information	11 (7.6)	11 (7.6)	35 (24.3)	33 (22.9)	54 (37.5)	4 (3–5)
3. Considering patient’s physical, socioeconomic, and emotional conditions while providing PC	8 (5.6)	5 (3.5)	37 (25.7)	43 (29.9)	51 (35.4)	4 (3–5)
4. Reviewing patient’s prescription or medication profile to determine possible DTRPs	2 (1.4)	7 (4.9)	41 (28.5)	42 (29.2)	52 (36.1)	4 (3–5)
5. Counselling patient to prevent potential DTRPs and to promote appropriate use of medicine	1 (0.7)	0	14 (9.7)	39 (27.1)	90 (62.5)	5 (4–5)
6. Resolving DTRPs of patient (e.g., Referring patient to doctor or communicating with doctor to resolve identified DTRPs)	3 (2.1)	9 (6.3)	41 (28.5)	48 (33.3)	43 (29.9)	4 (3–5)
7. Counselling patient on non-pharmacological management of their illness	3 (2.1)	16 (11.1)	47 (32.6)	38 (26.4)	40 (27.8)	4 (3–5)
8. Referring patients to doctor whenever necessary for further examination	3 (2.1)	10 (6.9)	28 (19.4)	48 (33.3)	55 (38.2)	4 (3–5)
9. Monitoring adverse effects of medicine	12 (8.3)	30 (20.8)	43 (29.9)	25 (17.4)	34 (23.6)	3 (2–4)
10. Monitoring patient’s treatment progress to assure achievement of therapeutic goal	7 (4.9)	29 (20.1)	34 (23.6)	40 (27.8)	34 (23.6)	4 (2.25–4)

*DTRPs* drug therapy-related problems (*any unwanted incident related to medication therapy that actually or potentially affects the desired goals of treatment*), *PC* pharmaceutical care.

## Detection of drug therapy related problems (DTRP) by the pharmacists

On an average, 134 pharmacists (93.1%) identified 1–100 errors related to drug dose, frequency, and duration; 135 (93.8%) pharmacists identified an equal range of errors related to drug name, dosage form and strength; 125 (86.8%) pharmacists identified equal errors on drug-drug interactions every month (Table 6).

**Table 6:** PC practices concerning DTRP identification.

Study variables	Frequency (%)
Errors on drug dose, frequency, and duration* (mean ± SD: 26.21 ± 51.27)	
0	3 (2.1)
1–100	134 (93.1)
101–200	6 (4.2)
401+	1 (0.7)
Total	144 (100)
**Errors on drug name, dosage form and strength (mean ± SD: 28.31 ± 61.76)**	
0	4 (2.8)
1–100	135 (93.8)
101–200	2 (1.4)
201–300	2 (1.4)
501+	1 (0.7)
Total	144 (100)
**Errors on drug-drug interaction (mean ± SD: 15.79 ± 29.10)**	
0	18 (12.5)
1–100	125 (86.8)
201+	1 (0.7)
Total	144 (100)
**Errors on adverse drug reactions (mean ± SD: 11.34 ± 15.89)**	
0	24 (16.7)
1+	120 (83.3)
Total	144 (100)

*Errors on drug dose refer to mistakes in writing dose of medicine or did not write it (either omission and commission type).

*Errors on frequency refers to mistakes in writing frequency/regimen of medicine or did not write it (either omission and commission type).

*Errors on duration refer to mistakes in drug duration writing or did not write it (either omission and commission type).

### Barriers to providing pharmaceutical care

Maximum pharmacists (82, 56.9%) agreed that patients never asked for pharmaceutical care from them, and an equal number agreed that medicine practice and policy were oriented towards dispensing only. Similarly, 85 (59%) agreed that supportive practice guidelines were lacking nationally (Table 7).

**Table 7:** Constructs related to barriers in providing pharmaceutical care (n: 144).

Constructs	Frequency (%)					Median (IQR) scores
	Strongly disagree	Disagree	Not sure	Agree	Strongly agree	
1. There is a lack of support from other health professionals toward pharmaceutical care	2 (1.4)	9 (6.3)	20 (13.9)	73 (50.7)	40 (27.8)	4 (4–5)
2. The co-ordination between pharmacists, doctors and other health professionals is poor	5 (3.5)	15 (10.4)	5 (3.5)	72 (50)	47 (32.6)	4 (4–5)
3. Patient is unable (due to illiteracy, unawareness or other reasons) to understand pharmaceutical care instructions	1 (0.7)	9 (6.3)	12 (8.3)	83 (57.6)	39 (27.1)	4 (4–5)
4. There is a lack of demand for and acceptance of pharmaceutical care by the patient	1 (0.7)	6 (4.2)	27 (18.8)	82 (56.9)	28 (19.4)	4 (4–4)
5. There is a lack of support from pharmacy owners or hospital administrators toward providing pharmaceutical care	2 (1.4)	13 (9)	13 (9)	75 (52.1)	41 (28.5)	4 (4–5)
6. There is a lack of supportive pharmaceutical care practice guideline	4 (2.8)	6 (4.2)	7 (4.9)	85 (59)	42 (29.2)	4 (4–5)
7. There is insufficient opportunity for pharmacists to interact closely with patients	2 (1.4)	9 (6.3)	17 (11.8)	69 (47.9)	47 (32.6)	4 (4–5)
8. Medicine practice and policy are more oriented toward medicine dispensing	1 (0.7)	4 (2.8)	6 (4.2)	82 (56.9)	51 (35.4)	4 (4–5)
9. Inadequate training is provided to pharmacist in providing pharmaceutical care	2 (1.4)	4 (2.8)	8 (5.6)	75 (52.1)	55 (38.2)	4 (4–5)
10. Pharmacists have inadequate therapeutic knowledge in resolving drug therapy-related problems	5 (3.5)	30 (20.8)	20 (13.9)	65 (45.1)	24 (16.7)	4 (3–4)
11. The education in the current pharmacy curriculum is inadequate to equip pharmacists to provide pharmaceutical care	3 (2.1)	15 (10.4)	12 (8.3)	59 (41)	55 (38.2)	4 (4–5)
12. Pharmacists lack skill in effective communication	9 (6.3)	43 (29.9)	23 (16)	57 (39.6)	12 (8.3)	3 (2–4)
13. Pharmacists lack skill in appropriate documentation	10 (6.9)	49 (34)	17 (11.8)	51 (35.4)	17 (11.8)	3 (2–4)
14. The attitude of pharmacists toward pharmaceutical care is inappropriate	12 (8.3)	44 (30.6)	32 (22.2)	49 (34)	7 (4.9)	3 (2–4)
15. Pharmacists lack self-confidence	15 (10.4)	54 (37.5)	16 (11.1)	48 (33.3)	11 (7.6)	3 (2–4)
16. Pharmacists lack motivation	14 (9.7)	23 (16)	13 (9)	69 (47.9)	25 (17.4)	4 (2–4)
17. There is lack of compensation or reimbursement to pharmacists for providing pharmaceutical care	5 (3.5)	10 (6.9)	13 (9)	76 (52.8)	40 (27.8)	4 (4–5)
18. There is a lack of appropriate computerized electronic system for maintaining the patients’ medical record	6 (4.2)	24 (16.7)	11 (7.6)	74 (51.4)	29 (20.1)	4 (3–4)
19. There is a lack of appropriate computerized electronic system for medication assessment support	4 (2.8)	21 (14.6)	13 (9)	75 (52.1)	31 (21.5)	4 (3–4)
20. There is a lack of trained pharmacist to provide pharmaceutical care	11 (7.6)	18 (12.5)	8 (5.6)	73 (50.7)	34 (23.6)	4 (3–4)
21. There is insufficient pharmacist manpower	8 (5.6)	23 (16)	14 (9.7)	53 (36.8)	46 (31.9)	4 (3–5)
22. Pharmacists lack access to the patient medical record	3 (2.1)	26 (18.1)	18 (12.5)	76 (52.8)	21 (14.6)	4 (3–4)
23. There is insufficient time to provide pharmaceutical care	4 (2.8)	26 (18.1)	16 (11.1)	66 (45.8)	32 (22.2)	4 (3–4)
24. There is lack of separate counselling area for patient’s privacy	4 (2.8)	4 (2.8)	7 (4.9)	68 (47.2)	61 (42.4)	4 (4–5)
25. There is lack of access to objective drug information sources	1 (0.7)	15 (10.4)	13 (9)	80 (55.6)	35 (24.3)	4 (4–4)

*DTRPs* drug therapy-related problems, *PC* pharmaceutical care.

### Perception, practice and barrier scores among different subgroups of respondents

The perception scores were significantly different among subgroups of respondents with different work experiences (p value 0.048), and practice scores differed based on the presence or absence of training in PC (p value 0.017) (Table 8).

**Table 8:** Scores of perception, practice and barriers to pharmaceutical care among subgroups of respondents.

Items	Perception score		Practice score		Barriers score	
	Total median score (IQR)	p value	Total median score (IQR)	p value	Total median score (IQR)	p value
**Gender**						
Male	26 (24–28)	0.448	37 (32–42)	0.426	96 (86–102)	0.659
Female	26 (25–28)		37 (34–45)		91.5 (85–103)	
**Age (in years)**						
22–31	26 (24–28)	0.660	37 (33–43)	0.434	94 (86–102)	0.428
32–41	27 (24–29)		36 (32.5–40)		97 (89.5–104.5)	
**Qualification**						
MPharm	26 (24.5–29)	0.436	36 (33.5–40)	0.746	94.5 (88–102.5)	0.875
PharmD	26 (25.5–28)		37 (34.5–41)		97 (84.5–101.5)	
BPharm	26 (24–28)		38 (33–43)		94 (86–102)	
**Work experience (years)**						
Less than 0.1	22 (22–22)	**0.048**	41 (40–42)	0.689	80 (79–81)	0.261
0.1–10.0	26 (25–28)		37 (33–43)		96 (86–103)	
10.1–20.0	24 (22.5–26.5)		37.5 (30–41.5)		92 (1–97)	
**Training in PC**						
No	26 (25–28)	0.960	36.5 (32–41.5)	**0.017**	95 (87.5–101.5)	0.909
Yes	26 (24–28)		39 (35–44)		94 (84.5–104)	
**Location of pharmacy**						
Private Hospital	26 (24–28)	0.934	37 (32–43)	0.217	93 (84–102)	0.530
Community/NGO hospital	26 (23–29)		42 (35–46)		100 (91–104)	
Government hospital	26 (24–28)		37 (34–40)		95 (88–102)	
**Working hour**						
35+	26 (24–28)	0.680	37 (33.5–43)	0.664	94 (86–102)	0.066
**Service provision**						
Outpatients only	26 (24.5–29)	0.907	37 (31.5–43)	0.625	101 (88–103.5)	0.641
Both outpatients and inpatients	26 (24–28)		37 (34–43)		94 (86–102)	
Inpatients only	26 (24–27.5)		41.5 (36–46)		93.5 (84.5–106)	
**Average number of pharmacists/shift**						
≤ 1	26 (25–28)	0.547	37 (34–42)	0.904	98 (88.5–102)	0.345
1–5	26 (25–28)		37 (32.5–44)		94 (86–102.5)	
6–9	26 (23–28)		37 (35–38)		93 (85–99)	
10–13	25 (23–28)		33 (32–41.5)		89.5 (89–90)	

*IQR* interquartile range (expressed as Q1–Q3).

Mann–Whitney U test was used for dichotomous variables and the Kruskal Wallis test for variables with three or more than three responses.

Significant values are in [bold].

### Correlation analysis of perception-related constructs and other variables

Kendall’s correlations were highly significant between constructs C1 and C2, C1 and C3, C2 and C3, C3 and C5, and C4 and C5, with a p value < 0.001 in each case, whereas there was no significant correlation of qualification with experience (p value 0.681), site of work (p value 0.386) and working hours per week (p value 0.153). Similarly, experience did not have a significant correlation with the site of work (p value 0.149) and working hours (p value 0.855) (Table 9).

**Table 9:** Correlation among different perception-related constructs and other variables.

Constructs/variables	τ (p value)											
	C1	C2	C3	C4	C5	C6	Qualification	Experience (In years)	Site of work	Working hours per week	Age (in years)	Number of daily prescriptions handled
C1	–	0.287**	0.430**	0.134 (0.088)	0.213* (0.009)	− 0.182* (0.017)	0.041 (0.610)	− 0.027 (0.737)	− 0.130 (0.098)	0.092 (0.263)	− 0.032 (0.691)	− 0.055 (0.496)
C2		–	0.434**	− 0.036 (0.634)	0.222*(0.004)	− 0.061 (0.405)	− 0.025 (0.740)	− 0.117 (0.136)	− 0.019 (0.802)	0.114 (0.150)	0.011 (0.886)	− 0.131 (0.092)
C3			–	0.257* (0.001)	0.370**	0.192* (0.012)	− 0.039 (0.631)	− 0.093 (0.259)	− 0.047 (0.550)	− 0.062 (0.455)	− 0.033 (0.685)	− 0.042 (0.602)
C4				–	0.395**	0.136 (0.068)	− 0.121 (0.122)	0.066 (0.409)	− 0.005 (0.949)	− 0.070 (0.381)	0.009 (0.908)	0.102 (0.195)
C5					–	0.034 (0.656)	− 0.073 (0.364)	− 0.007 (0.932)	0.057 (0.471)	− 0.075 (0.364)	0 (0.995)	0.064 (0.426)
C6						–	− 0.104 (0.166)	− 0.117 (0.129)	− 0.056 (0.450)	0.026 (0.740)	0.171* (0.027)	0.023 (0.765)
Qualification							–	− 0.033 (0.681)	− 0.067 (0.386)	0.116 (0.153)	− 0.195 (0.016)	0.008 (0.916)
Experience (in years)								–	0.114 (0.149)	0.015 (0.855)	0.467**	0.062 (0.444)
Site of work									–	− 0.090 (0.259)	0.095 (0.230)	0.154 (0.050)
Working hours per week										–	0.035 (0.674)	0.034 (0.674)
Age (in years)											–	0.114 (0.162)

C1: Patient’s medications should be reviewed to prevent medicine-related errors and promote appropriate use of medications.

C2: All patients receiving medicines require PC.

C3: PC can improve patient’s treatment or health outcome.

C4: Pharmacists are professionally skilled HPs in providing PC.

C5: Pharmacists are responsible for the identification, prevention and resolution of MRPs.

C6: Continuing pharmacy education is NOT essential to equip pharmacists to provide PC.

C: Construct; τ: Kendall’s correlation (Tau).

**p value < 0.001.

### Correlation analysis of practice-related various constructs and other variables

Kendall’s correlations were highly significant between constructs C1 and C2, C1 and C3, C1 and C4, C1 and C6, C1 and C7, C1 and C8, C1 and C9, C1 and C10, and many other constructs, with p value < 0.001 in each case (Table 10).

**Table 10:** Correlation among practice-related various constructs and other variables.

Constructs/variables	τ (p value)															
	C1	C2	C3	C4	C5	C6	C7	C8	C9	C10	Qualification	Experience (in years)	Site of work	Working hours per week	Age (in years)	Number of daily prescriptions handled
C1	–	0.342**	0.289**	0.436**	0.227* (0.002)	0.304**	0.249**	0.295**	0.300**	0.313**	− 0.009 (0.903)	− 0.095 (0.212)	− 0.047 (0.519)	0.063 (0.409)	− 0.149 (0.051)	0.162 (0.032)
C2		–	0.279**	0.285**	0.222* (0.003)	0.062 (0.382)	0.058 (0.404)	0.149* (0.035)	0.191* (0.006)	0.222* (0.001)	0.030 (0.686)	− 0.031 (0.684)	− 0.030 (0.682)	− 0.003 (0.970)	− 0.109 (0.152)	0.160 (0.032)
C3			–	0.243* (0.001)	0.231*(0.002)	0.188* (0.008)	0.301**	0.195* (0.006)	0.200* (0.004)	0.263**	0.043 (0.564)	− 0.004 (0.962)	− 0.019 (0.796)	0.079 (0.308)	− 0.116 (0.129)	0.068 (0.366)
C4				–	0.427**	0.296**	0.215* (0.002)	0.175* (0.015)	0.239* (0.001)	0.285**	− 0.029 (0.698)	− 0.006 (0.937)	− 0.051 (0.490)	0.083 (0.282)	− 0.032 (0.676)	0.064 (0.400)
C5					–	0.108 (0.148)	0.255* (0.001)	0.201* (0.007)	0.268**	0.314**	0.058 (0.463)	− 0.039 (0.630)	0.000 (0.996)	0.085 (0.293)	− 0.109 (0.176)	− 0.011 (0.892)
C6						–	0.300**	0.342**	0.319**	0.300**	0.021 (0.783)	0.102 (0.182)	0.027 (0.712)	0.077 (0.320)	0.051 (0.503)	0.067 (0.375)
C7							–	0.304**	0.303**	0.314**	0.066 (0.377)	− 0.018 (0.818)	0.050 (0.497)	0.057 (0.460)	− 0.063 (0.408)	− 0.044 (0.557)
C8								–	0.319**	0.258**	− 0.001 (0.990)	− 0.092 (0.231)	− 0.067 (0.368)	0.088 (0.254)	− 0.109 (0.157)	0.115 (0.131)
C9									–	0.566**	0.009 (0.898)	− 0.223** (0.003)	− 0.044 (0.543)	0.016 (0.833)	− 0.107 (0.152)	0.022 (0.764)
C10										–	0.051 (0.488)	− 0.030 (0.693)	− 0.048 (0.509)	0.036 (0.638)	− 0.028 (0.706)	0.026 (0.721)
Qualification											–	#	#	#	#	#
Experience (in years)												–	#	#	#	#
Site of work													–	#	#	#
Working hours per week														–	#	#
Age (in years)															–	#

C1: Enquiring about and reviewing patient’s medical and medicine records to decide if any intervention or recommendation must be made.

C2: Documenting patient’s clinical and medication information record.

C3: Considering patient’s conditions (physical, social, emotional, economic etc.) while providing pharmaceutical care.

C4: Reviewing the patient’s prescription or medication profile to determine possible drug therapy-related problems or errors.

C5: Counselling the patient to prevent potential drug-therapy related problem and to promote appropriate use of medicine.

C6: Resolving the drug therapy-related problem of patient. (e.g., referring the patient to doctor or communicating with the doctor to resolve the identified drug therapy-related problem).

C7: Counselling the patient on non-pharmacological management of their illness.

C8: Referring patients to doctor whenever necessary for further examination.

C9: Monitoring adverse effects or reactions of medicine in patient.

C10: Monitoring patient’s treatment progress to assure the achievement of therapeutic goal.

C: Construct; τ: Kendall’s correlation (Tau); #: as in Table 7; **p value < 0.001.

## Discussion

Pharmacy practice in Nepal still has only a minimal patient focus. There have been initiatives to improve the situation of the pharmacy profession in the country, with more focus on patient-centeredness. Two initiatives to promote the pharmacy profession in Nepal include establishing a master’s in pharmacy program in pharmaceutical care at Kathmandu University in 2000 with alumni working in hospitals, academic and regulatory affairs, and drafting good pharmacy practice (GPP) guidelines in November 2005^49^. Unfortunately, during the past two decades, the country has witnessed significant challenges such as an armed insurgency, political instability, and a major earthquake in 2015, all linked with poor employment, instability, and brain drain of qualified/skilled health workers^49^. These changes delayed the implementation of the draft GPP guidelines at the hospital level though there have been some initiatives from the government to improve the rational use of medicines in hospitals. The hospitals either outsource pharmacies to private parties on monthly rent or run as a minimalistic pharmacy setup with minimal space, infrastructure, and human resources, focusing only on procurement, storage, and selling of medicines and non-medical supplies^22^. There have been no studies assessing the patient care-related contribution of pharmacists in hospitals. The present study, probably the first of its kind in Nepal, reported a positive perception among hospital pharmacists and a reasonably good level of practice and many barriers encountered in offering PC services.

### Perception regarding providing pharmaceutical care

Unfortunately, PC activities were absent in most hospitals in Nepal, with a few exceptions wherein self-motivated pharmacists offer these services individually or at their department level. Contrary to the conventional wisdom, the present study demonstrated a positive perception linked to a willingness to perform PC services and pharmacist’s work experience. The present study findings are similar to those reported by community pharmacists in China^36^, USA^50^, and Nigeria^51^. The perception part of the study questionnaire had six questions, and all responses had a median score of 4 or 5, suggesting a positive perception. However, it is essential to note that four respondents strongly disagreed with the statement ‘*Patient’s medications should be reviewed to prevent medicine-related errors and promote appropriate use of medications*, which shows pharmacists restricting themselves to traditional product-oriented roles and not offering patient care services. Pharmacists are expected to possess important skills such as communication, history taking, and physical assessment to offer PC services. In the present research, 7.6% of the respondents strongly disagreed that pharmacists in Nepal are professionally skilled in providing pharmaceutical care. Considering all the respondents have a minimum qualification of BPharm, the findings show the need for educational reforms and continuing professional education to train the pharmacists towards PC. Shrestha et al.^19^ recommended major changes in pharmacy education and focus on patient care education. In the present study, the pharmacists’ perception of PC was not influenced by demographic parameters other than the years of service. This finding suggests a general agreement among all pharmacists on the importance of PC services. The duration of service can naturally impact the pharmacist's attitude towards PC as more patient contact can help accept the professional roles.

### Current practice of pharmaceutical care among hospital pharmacists

In line with the positive perception, the pharmacists also had a good practice related to PC services. However, it is noteworthy that this research was based on a self-reported survey, and the actual practice in the hospitals in terms of the quality of service was not verified by the researchers. The present study findings disagree with research from community pharmacies in Jordan^52^, Malaysia^53^, and hospitals in Pakistan^54^, wherein authors reported very limited or no PC services offered by community pharmacists. The location was different, being a hospital pharmacy in our study and a community pharmacy in others. These positive changes noted in the present study signify the recent changes in pharmacy practice in the country. The present study finding shows pharmacists' poor documentation of patients’ clinical and medication information, which can eventually be a barrier to practice. The American Society of Health-System Pharmacists (ASHP) recommended that pharmacists be authorized to write inpatient medication records to document their assessments, conclusions, and recommendations on drug therapy^10^. However, this has not been implemented in Nepal. To offer patient care, the pharmacists should at least have access to medical records, a process that is largely missing in Nepal’s hospital pharmacies, although most hospitals have well-equipped computerized billing software. This gap requires intervention to improve pharmacists’ access to medical records and competence in interpreting patient data.

Further, the results showed a relatively low score for the statement ‘Reviewing patient’s prescription or medication profile to determine possible DTRPs. Studies from different countries have reported that pharmacists’ prescription reviews can help identify and mitigate DTRPs^55–57^. Identification and mitigation of DTRPs may be improved by providing more training to the pharmacists and improving access to drug information resources. Electronic databases can also help screen the prescriptions and detect DTRPs. While resolving DTRPs, one might require referring the patients to their physician, which most pharmacists did in reported studies. Though the pharmacists referred the patients to physicians, the extent of interprofessional collaboration (IPC) between pharmacists and physicians is not well studied in Nepal. Since lack of IPC can be a major barrier in providing PC, more research is needed.

PC practice is primarily influenced by training related to PC undergone by the pharmacists during their academic curriculum. At this point, it is worth mentioning that the curricula for pharmacists are inadequate to train the graduates in offering PC services. In addition, there are also challenges in offering experiential learning to the graduates, which lead to pharmacists being incompetent in patient care^19^. The present study findings also showed the existence of good counselling practices focusing on DTRPs prevention and non-pharmacological management of diseases, which is a welcome development. However, it was worth noting that pharmacists did not contribute much to adverse drug reactions (ADR) reporting and monitoring patients’ treatment progress to assure therapeutic goals. This can be improved only with proper education and training. Since underreporting of ADRs is considered a significant barrier in the current national pharmacovigilance program^24,58^, proper education and training of pharmacists can be valuable to improve the reporting rates and prevent their recurrence.

### Perceived barriers to providing pharmaceutical care

The practice of PC can be primarily influenced by certain factors which can be modified to offer potential benefits. The common barriers to practicing pharmaceutical care reported in the literature are lack of pharmacist skills, lack of support from management, busy schedule, lack of incentives, etc.^8,38,43,44,59^. In the present study, the pharmacists reported multiple barriers (Table 8), like those reported in the literature^8,38,43,44,59^. These barriers encountered by the pharmacists were not influenced by their demography. There is a lack of support from other health professionals and poor coordination. The cooperation of other health professionals, especially physicians, is essential for promoting patient care and health outcomes.

On the contrary, a study from Kuwait reported physician agreement on pharmacists' contribution in managing ADRs, improving adherence, dosage adjustment, offering advice on drug interactions, and providing drug information (DI) to physicians^60^. The barrier noticed in the present study may be addressed by incorporating interprofessional education in the health professional curricula. Other barriers related to patients are poor literacy rates and lack of demand for PC. Health literacy is important for patient adherence, and in Nepal, there is a low literacy rate, especially in rural areas, which can certainly be a barrier. This can be overcome by offering customized patient-friendly educational materials by pharmacists.

Majority of hospital pharmacies in the country are outsourced by hospital management and are run as mere business entities. However, hospitals require their own hospital pharmacy according to hospital pharmacy guidelines^61^. This requires stringent regulatory intervention by the national drug regulatory body, i.e., DDA, to stop the practice of outsourcing pharmacies immediately. Pharmacists felt a lack of supportive PC practice guidelines in practising PC. More awareness needs to be created of the current GPP guidelines among pharmacists, and special training on GPP adherence may be conducted. Two studies conducted among community pharmacists reported poor compliance with GPP guidelines^26,62^. Though the setting is different, this may also have implications for pharmacy practice in hospital pharmacies.

Pharmacists perceived major barriers concerning their education, competency, and training. These barriers can be overcome only by improving pharmacy education and equipping future pharmacists with more competencies related to patient care. Since most pharmacies are more business-focused, one can expect a lack of human resources leading to a busy work schedule that naturally limits offering PC services. Pharmacists in Nepal also felt a lack of compensation as a barrier to PC services, similar to pharmacists from Nigeria^63^. The layout of the pharmacies is also crucial to examine patients in a private area and offer counselling which is mostly lacking in Nepal. The GPP guidelines of Nepal, however, emphasize layout requirements for pharmacies^49^.

### Recommendations

The study findings recommend that more pharmacists are trained in patient care processes such as history taking, physical assessment, DTRPs identification and mitigation, and patient counselling. In addition, the pharmacy curricula must be critically examined and, if necessary, should be updated to offer competencies in PC. Furthermore, the pharmacies should ensure adequate human resources and pharmacy facilities to offer PC services. Outsourcing of pharmacies to private parties should be stopped, and hospitals should run their pharmacies. The GPP guideline, which is in the draft version, should be implemented without delay, and the implementation should be assessed periodically. Along with that, despite the hospital pharmacists finding an unfavorable situation to initiate PC, they should at least attempt to provide pharmaceutical care services such drug information, medication adherence monitoring and counseling on appropriate use, consulting or recommending patients to prescriber when they encounter any medication errors and drug interactions, and creating awareness on adverse drug reaction among patient and prescribers.

### Strengths and limitations of the study

This is the first study to assess the pharmacists’ perception, practice, and barriers to PC services in Nepal. Furthermore, it is a nationwide study representing pharmacists in the entire country. Along with significant strengths, the study also has some limitations. First, the study used a convenient sampling method, and the authors could not achieve the requisite sample size. Secondly, the study only concentrated on pharmacists working in a hospital pharmacy setting. Therefore, it only represents the pharmaceutical care practices of hospital pharmacies. Thirdly, the study used an online Google survey link for data collection and shared the link through professional networks to reach only pharmacy professionals rather than sharing publicly on social media. Finally, this research was conducted during the peak period of COVID-19 pandemic prior to vaccine rollout, which might have limited pharmacists offering patient care services, thus influencing their responses. There is also a possibility of the Dunning–Kruger effect^64^ influencing the pharmacists’ responses to questions.

## Conclusions

Hospital pharmacists who participated had a positive perception of providing pharmaceutical care. However, PC is not commonly practiced in hospital pharmacies. Significant barriers in providing PC were identified. Pharmacists believed that they might not have the requisite training to provide PC, access to patient records remained poor and commercial interests dominated hospital pharmacies. Adequate space, proper layout and adequate human resources are important to providing PC. Further studies, especially in the eastern and western provinces, are required. Similar studies may be considered in community pharmacies.

## Supplementary Information

Supplementary Information 1.

Supplementary Information 2.

## Data Availability

The datasets used and/or analyzed during the current study available from the corresponding author on reasonable request.
