# Supplementary material for: A nationwide exploratory survey assessing perception, practice, and barriers toward pharmaceutical care provision among hospital pharmacists in Nepal
Source: Sci Rep. 2022 Oct 5;12:16590. doi: 10.1038/s41598-022-16653-x (PMC9532804; doi:10.1038/s41598-022-16653-x)
Supplement: Supplementary file 1 — Supplementary Information 1. [file 41598_2022_16653_MOESM1_ESM.docx]

**A nationwide exploratory survey assessing perception, practice, and barriers toward pharmaceutical care provision among hospital pharmacists in Nepal**

**Authors**

Rajeev Shrestha^1*^, Subish Palaian^2^, Binaya Sapkota^3*^, Sunil Shrestha^4*^, Asmita Priyadarshini Khatiwada^4^, Pathiyil Ravi Shankar^5^

**Data Collection tool**

**PARTICIPANT’S DEMOGRAPHIC AND WORK-RELATED INFORMATION**

**1. Age…**……  **2. Gender:** Male/Female/Others

**3. Highest degree:** Bachelor of Pharmacy/PharmD/Master of Pharmacy/PhD

**4. Work experience in years: ………….**

**5. Received training in pharmaceutical care**: Yes/No

**6. Current place of work:** Government hospital pharmacy/Private hospital pharmacy/Community or NGO Hospital Pharmacy

7. **Number of hours the pharmacy is open daily:** ……………….

**8. Pharmacy provides services to**: Inpatients only or Outpatient only or both

**9. Number of beds in the hospital**: ………………………………….….

**10. Location of hospital (e.g. Bhaktapur):**.……………………………..…

**11. Your average working hours per week (e.g. 42 hours): ……………...**

**12. Approximate (average) number of prescriptions handled by you daily:…….……**

**14. Average number of pharmacists during each shift:…………………**

**PERCEPTION RELATED QUESTIONS**

| Perception (*Please tick on your answer*) | Strongly Disagree | Disagree | Not Sure | Agree | Strongly Agree |
| --- | --- | --- | --- | --- | --- |
| 15. Patient’s medications should be reviewed in order to prevent medicine-related errors and promote appropriate use of medications |  |  |  |  |  |
| 16.All patients receiving medicines require pharmaceutical care service |  |  |  |  |  |
| 17. Pharmaceutical care can improve patient’s treatment or health outcome |  |  |  |  |  |
| 18. Pharmacists are professionally skilled health personnel in providing pharmaceutical care |  |  |  |  |  |
| 19. Pharmacists are responsible for identification, prevention and resolution of medicine-related problems |  |  |  |  |  |
| 20. Continuing pharmacy education is NOT essential to equip pharmacists to provide pharmaceutical care |  |  |  |  |  |

**PRACTICE RELATED QUESTIONS**

| Practice (*Please tick on your answer*) | Never | Rare | Sometimes | Usually | All the time |
| --- | --- | --- | --- | --- | --- |
| **Monitoring and examining patient** |  |  |  |  |  |
| 21. Enquiring about and reviewing patient’s medical and medicine records to decide if any intervention or recommendation must be made |  |  |  |  |  |
| 22. Documenting patient’s clinical and medication information record. |  |  |  |  |  |
| 23. Considering patient’s conditions (physical, social, emotional, economic etc.) while providing pharmaceutical care. |  |  |  |  |  |
| **Identification and prevention of drug therapy-related problem (***Drug therapy related problem means any unwanted incident related to medication therapy that actually or potentially affects the desired goals of treatment. For example high dose or low dose of drug administration, adverse effects etc***)** |  |  |  |  |  |
| 24. Reviewing the patient’s prescription or medication profile to determine possible drug therapy-related problems or errors. |  |  |  |  |  |
| 25. Counseling the patient to prevent potential drug-therapy related problem and to promote appropriate use of medicine |  |  |  |  |  |
| **Management of Drug-therapy-related problem** |  |  |  |  |  |
| 26. Resolving the drug therapy-related problem of patient. (e.g. Referring the patient to doctor or communicating with the doctor to resolve the identified drug therapy-related problem) |  |  |  |  |  |
| 27. Counseling the patient on non-pharmacological management of their illness |  |  |  |  |  |
| 28. Referring patients to doctor whenever necessary for further examination. |  |  |  |  |  |
| **Drug therapy monitoring** |  |  |  |  |  |
| 29. Monitoring adverse effects or reactions of medicine in patient |  |  |  |  |  |
| 30. Monitoring patient’s treatment progress to assure the achievement of therapeutic goal. |  |  |  |  |  |
| 31. Documentation of detected errors and interventions ( open question) |  | | | | |
| Average number of errors related to drug dose, frequency and duration issues per month |  | | | | |
| Average number of errors related to drug name, dosage form and strength issues per month |  | | | | |
| Average number of errors related to drug–drug interaction per month |  | | | | |
| Average number of errors related to adverse drug reactions per month |  | | | | |

**BARRIER RELATED QUESTIONS**

| Barriers (*Please tick on your answer*) | Strongly Disagree | Disagree | Not Sure | Agree | Strongly Agree |
| --- | --- | --- | --- | --- | --- |
| **Patients and health care providers related** |  |  |  |  |  |
| 32. There is a lack of support from other health professionals toward pharmaceutical care |  |  |  |  |  |
| 33. The co-ordination between pharmacists, doctors and other health professionals is poor. |  |  |  |  |  |
| 34. Patient is unable (due to illiteracy, unawareness or other reasons) to understand pharmaceutical care instructions. |  |  |  |  |  |
| 35. There is a lack of demand for and acceptance of pharmaceutical care by the patient |  |  |  |  |  |
| **Legal and social factors related** |  |  |  |  |  |
| 36. There is a lack of support from pharmacy owners or hospital administrators toward providing pharmaceutical care |  |  |  |  |  |
| 37. There is a lack of supportive pharmaceutical care practice guideline |  |  |  |  |  |
| 38. There is insufficient opportunity for pharmacists to interact closely with patients. |  |  |  |  |  |
| 39. Medicine practice and policy are much oriented toward medicine dispensing. |  |  |  |  |  |
| **Education, knowledge and skills of Pharmacist related** |  |  |  |  |  |
| 40. Inadequate training is provided to pharmacist in providing pharmaceutical care |  |  |  |  |  |
| 41. Pharmacists have inadequate therapeutic knowledge in resolving drug therapy-related problems |  |  |  |  |  |
| 42. The education in the current pharmacy curriculum is inadequate to provide pharmaceutical care |  |  |  |  |  |
| 43. Pharmacists lack skill in effective communication |  |  |  |  |  |
| 44. Pharmacists lack skill in appropriate documentation |  |  |  |  |  |
| **Initiation related** |  |  |  |  |  |
| 45. The attitude of pharmacists toward pharmaceutical care is inappropriate |  |  |  |  |  |
| 46. Pharmacists lack self-confidence |  |  |  |  |  |
| 47. Pharmacists lack motivation |  |  |  |  |  |
| 48. There is lack of compensation or reimbursement to pharmacists for providing pharmaceutical care |  |  |  |  |  |
| **Resource and Facility related** |  |  |  |  |  |
| 49. There is a lack of appropriate computerized electronic system for maintaining the patients’ medical record |  |  |  |  |  |
| 50. There is a lack of appropriate computerized electronic system for medication assessment support |  |  |  |  |  |
| 51. There is a lack of trained pharmacist to provide pharmaceutical care |  |  |  |  |  |
| 52. There is insufficient pharmacist manpower |  |  |  |  |  |
| 53. Pharmacists lack access to the patient medical record |  |  |  |  |  |
| 54. There is insufficient time to provide pharmaceutical care |  |  |  |  |  |
| 55. There is lack of separate counseling area for patient’s privacy |  |  |  |  |  |
| 56. There is lack of access to effective drug information sources |  |  |  |  |  |
| Any other barriers (Please specify) |  | | | | |
