# Supplementary material for: A nationwide exploratory survey assessing perception, practice, and barriers toward pharmaceutical care provision among hospital pharmacists in Nepal
Source: Sci Rep. 2022 Oct 5;12:16590. doi: 10.1038/s41598-022-16653-x (PMC9532804; doi:10.1038/s41598-022-16653-x)
Supplement: Supplementary file 2 — Supplementary Information 2. [file 41598_2022_16653_MOESM2_ESM.docx]

**Supplementary Table 1:** **Location of hospitals**

| Location | | Frequency (%) |
| --- | --- | --- |
| **Province (n,%)** | **Districts** |  |
| 1 (5,3.5) | Sunsari | 3 (2.1) |
|  | Morang | 2 (1.4) |
| 2 (5, 3.5) | Dhanusa | 3 (2.1) |
|  | Parsa | 2 (1.4) |
| 3 (75, 52.08) | Kathmandu | 39 (27.1) |
|  | Bhaktapur | 8 (5.6) |
|  | Chitwan | 9 (6.3) |
|  | Lalitpur | 13 (9.0) |
|  | Makwanpur | 3 (2.1) |
|  | Kavrepalanchowk | 2 (1.4) |
|  | Sindhuli | 1 (0.7) |
| 4 (21,14.6) | Kaski | 17 (11.8) |
|  | Baglung | 1 (0.7) |
|  | Lamjung | 1 (0.7) |
|  | Manang | 1 (0.7) |
|  | Tanahun | 1 (0.7) |
| 5 (23,16) | Dang | 2 (1.4) |
|  | Banke | 7 (4.9) |
|  | Palpa | 4 (2.8) |
|  | Rupandehi | 10 (6.9) |
| 6 (5,3.5) | Surkhet | 3 (2.1) |
|  | Rukum Paschim | 1 (0.7) |
|  | Dailekh | 1 (0.7) |
| 7 (10,6.9) | Doti | 2 (1.4) |
|  | Kailali | 4 (2.8) |
|  | Dadeldhura | 4 (2.8) |
| **Total** | | **144 (100)** |
